# Testing the Relationship between Human Occupancy in the Landscape and Tadpole Developmental Stress

**DOI:** 10.1371/journal.pone.0120172

**Published:** 2015-03-20

**Authors:** Paula C. Eterovick, Luís F. F. Bar, Jorge B. Souza, José F. M. Castro, Felipe S. F. Leite, Ross A. Alford

**Affiliations:** 1 Programa de Pós Graduação em Zoologia de Vertebrados, Pontifícia Universidade Católica de Minas Gerais, Belo Horizonte, Minas Gerais, Brazil; 2 Programa de Pós Graduação em Geografia—Tratamento da Informação Espacial, Pontifícia Universidade Católica de Minas Gerais, Belo Horizonte, Minas Gerais, Brazil; 3 Instituto de Ciências Biológicas e da Saúde, Universidade Federal de Viçosa, Campus Florestal, Florestal, Minas Gerais, Brazil; 4 College of Marine and Environmental Science, Centre for Tropical Biodiversity and Climate Change, James Cook University, Townsville, Queensland, Australia; Trier University, GERMANY

## Abstract

Amphibian population declines are widespread; the main causal factors are human related and include habitat fragmentation due to agriculture, mining, fires, and urban development. Brazil is the richest country in species of amphibians, and the Brazilian regions with the greatest amphibian diversity are experiencing relatively high rates of habitat destruction, but there are presently relatively few reports of amphibian declines. It is thus important to develop research methods that will detect deterioration in population health before severe declines occur. We tested the use of measurements of fluctuating asymmetry (FA) taken on amphibian larvae to detect anthropogenic stress. We hypothesized that greater human occupancy in the landscape might result in more stressful conditions for amphibians. We conducted this study at the Espinhaço mountain range in southeastern Brazil, using as a model an endemic species (*Bokermannohyla saxicola*, Hylidae). We chose two tadpole denticle rows and eye-nostril distance as traits for FA measurement. We measured percent cover of human-altered habitats in the landscape around tadpole sampling points and measured FA levels in sampled tadpoles. We found FA levels to differ among localities but found no relationship between human modification of the landscape and tadpole FA levels. Levels of FA in the traits we examined may not be strongly affected by environmental conditions, or may be affected by local variables that were not captured by our landscape-scale measures. Alternatively, populations may be genetically differentiated, affecting how FA levels respond to stress and obscuring the effects of anthropogenic disturbance.

## Introduction

Many studies have reported amphibian declines throughout the world [1–4]. Suggested causes include anthropogenic environmental alterations such as habitat fragmentation caused by agriculture expansion, cattle raising, mining, fires, and construction of human settlements and structures such as dams and roads [3, 5]. Other possible causes are consequences of human activities as well, such as climatic changes, acid rain, and the introduction of exotic species [6, 7]. Another cause is the recent widespread emergence of epidemics of the disease chytridiomycosis, caused by the chytrid fungus *Batrachochytrium dendrobatidis* Longcore, Pessier and Nichols, 1999. Anthropogenic impacts such as climate changes [8] and the international trade or transport of amphibians by humans [9, 10] are likely to be increasing the spread of this chytrid.

Amphibians are sensitive to environmental alterations, and can be strongly affected by pollutants, and by temperature and humidity changes due to their permeable skins and the complex life cycles of most species [11, 12]. Population declines can occur rapidly, so monitoring techniques that allow the detection of potential problems before they actually cause a decline are desirable to increase the chances of recovery [13]. Monitoring Developmental Instability (DI) is one such technique [14].

Developmental stability is the ability of a genotype to repeatedly express a single phenotype when development occurs under the same environmental conditions [15]. Deviations from the ideal phenotype arise due to random fluctuations in developmental processes (developmental noise) and are corrected by homeostatic mechanisms [16]. The mean level of these deviations at any time (the level of DI) reflects the balance between noise and correction [16]. Developmental instability is likely to increase in individuals exposed to high levels of stress during their development because stress can increase levels of developmental noise, decrease the functioning of homeostatic mechanisms, or both [16]. High levels of stress may interact with other factors to make amphibian populations more likely to suffer declines [17, 18].

For many bilateral traits it appears that the genetic program specifies perfect symmetry, so that deviations from this may reflect DI [15]. Divergences from perfect symmetry in a population can be classified as directional asymmetry, antisymmetry, or fluctuating asymmetry [19]. Directional asymmetry occurs when a character is consistently larger on one particular side. Antisymmetry occurs when a character is consistently larger on one side, but which side is larger differs among individuals [19–21]. Fluctuating asymmetry (FA) occurs when there is a symmetrical, unimodal distribution of differences between right and left sides of a character with a mean at perfect symmetry [19]. Directional asymmetry and antisymmetry may reflect genetic programming for asymmetry, but FA should be a measure of the balance between developmental noise and the mechanisms that correct it [16, 19]. FA is widely used to detect the influence of disturbances in species development, with special focus on human impacts [18, 22–26].

FA can vary during ontogeny, that is, individuals may present different asymmetry levels at different ages. Thus, FA reflects the recent development of the organism [16]. In anurans, high mortality rates have been observed in the larval stage [27, 28]. Thus, considering that individuals with high levels of DI may be selected against [13, 29, 30], we assumed that the detection of high levels of DI reflecting environmental impacts would be more likely in the larval stage. This would arise because more asymmetrical individuals (those with a lower capacity to control their development) may be more likely to die before they metamorphose (i.e., symmetrical individuals are likely to live longer [29]).

Brazil has 1026 described amphibian species [31]. Some are known to have declined, but there is a general lack of information on species natural history and few long term population studies [2]. Both the Cerrado and the Atlantic Forest are Brazilian amphibian hotspots, and both have experienced extensive destruction of natural habitats by humans [32]. Most reported cases of amphibian population declines refer to the Atlantic Forest [33–38]. The Cerrado region had its first cases recorded at Serra do Cipó, in the Espinhaço mountain range, southeastern Brazil [2].

Because many aspects of anthropogenic modification of habitats are likely to impose stress on amphibians [39], we hypothesized that the impact suffered by amphibians would be proportional to human occupancy in a montane meadow landscape (a species rich highland formation included in the Cerrado biome), as measured by the percentage of the surface modified by human activities. This effect should be strong in species with a tadpole stage that are likely to migrate between suitable terrestrial and aquatic habitats that constitute selected breeding sites [11]. For these species, human impacts are likely to result in both habitat loss and impairment of established migration routes [11]. Thus, we chose the treefrog *Bokermannohyla saxicola*, an endemic of montane meadow habitats, as a model.

*Bokermannohyla saxicola* (Bokermann, 1964) lives in highlands (mainly above 1000 m) and breeds in streams [40]. Highland species have repeatedly been reported to be the most sensitive and susceptible to declines in other localities [4, 8, 41, 42]. As a high elevation stream-associated species with an aquatic larval stage, *B*. *saxicola* might thus be particularly vulnerable to negative effects of human alteration of its natural habitat. The streams where this species has been recorded are relatively well preserved, probably because they occur in hilly rocky terrains that are not very attractive to humans for activities such as agriculture and cattle raising (FSFL, PCE, personal observation). We are searching for a preventive method to detect impacts that could cause amphibian declines and in order to be the most effective such methods should work as early as possible. Thus, a species that still lives in habitats with relatively little alteration is a good subject to test the efficacy of the method. We therefore hypothesized that levels of tadpole FA in *B*. *saxicola* populations should be positively correlated with the percentage of the adjacent landscape modified by humans.

## Materials and Methods

### Study site and data collection

*Bokermannohyla saxicola* is endemic to the Espinhaço Range and has a broad distribution within it [43–45]. This mountain chain is a geographical barrier between the biomes of Cerrado and Atlantic Forest [44]. Currently, 105 anuran species are recorded for the Espinhaço [44]; the distributions of 33 of these are restricted to this mountain range (28 in Minas Gerais state, including *B*. *saxicola*; [46]). Thus, the effects of human occupancy observed on *B*. *saxicola* may also be suffered by many other endemic anuran species.

**Fig 1:**
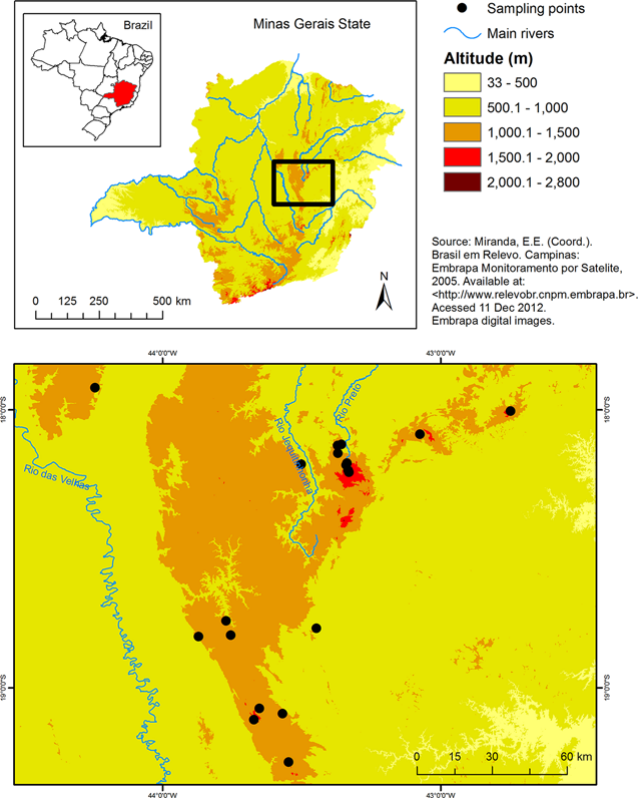
Sampling points of *Bokermannohyla saxicola* tadpoles in the Espinhaço Range, southeastern Brazil. Sampling points of *Bokermannohyla saxicola* tadpoles in the Espinhaço Range, southeastern Brazil, used for quantification of fluctuating asymmetry and anthropogenic modification of the landscape (measured as percent cover of human soil use classes) around sampling points.

We analyzed 249 tadpoles of *B*. *saxicola* in developmental stage 25 [47] colected from 2006 to 2008 in their natural habitats at 18 localities distributed in 9 municipalities throughout the core area of *B*. *saxicola* distribution (Table 1, Fig. 1). Developmental stage 25 is relatively long in tadpoles of *B*. *saxicola* and most of tadpole’s growth occurs in this stage [48]. Tadpoles attain low densities, even in streams where they are more abundant. Thus, for several localities, we were not able to find a large number of tadpoles within an area that could be considered one sampling point for the purposes of this study.

**Table 1:** Description of sampling points of *Bokermannohyla saxicola* tadpoles in the Espinhaço mountain range, southeastern Brazil.

N_ID	Locality	Municipality	Coordinates	N
1	**APA Serra Talhada**	Congonhas do Norte	S18 48 40.9 W43 45 16.7	29
2	**Ribeirão Congonhas**	Congonhas do Norte	S18 45 34.5 W43 46 14.9	10
3	**PE do Rio Preto (Éguas Stream 1)**	São Gonçalo do Rio Preto	S18 07 44.4 W43 22 12.0	3
4	**PE do Rio Preto (Éguas Stream 2)**	São Gonçalo do Rio Preto	S18 07 30.0 W43 21 25.2	2
5	**PE do Rio Preto (També Stream 1)**	São Gonçalo do Rio Preto	S18 13 30.0 W43 19 48.0	14
6	**PE do Rio Preto (També Stream 2)**	São Gonçalo do Rio Preto	S18 13 04.5 W43 19 58.7	6
7	**PE do Rio Preto (Serra Abóboras)**	São Gonçalo do Rio Preto	S18 11 56.4 W43 20 24.0	7
8	**APA Morro da Pedreira (Serra do Cipó)**	Santana do Riacho	S19 16 01.2 W43 32 48.5	1
9	**PE Serra Negra**	Itamarandiba	S18 00 18.3 W42 44 55.0	31
10	**Tabuleiro**	Conceição do Mato Dentro	S 19 05 36 W 43 34 07	10
11	**Fechados**	Santana de Pirapama	S 18 48 58 W 43 52 13	6
12	**Fazenda Calçada/ Pico do Breu**	Santana do Riacho	S 19 04 27 W 43 39 06	22
13	**Lapinha da Serra**	Santana do Riacho	S 19 06 55 W 43 40 16	3
14	**PE do Rio Preto**	Diamantina	S 18 11 32 W 43 20 11	4
15	**Conselheiro Mata**	Buenópolis	S 17 55 17 W 44 14 32.4	8
16	**Distrito de Pedra Menina**	São Gonçalo do Rio Preto	S 18 05 18 W 43 04 29	28
17	**APA Felício dos Santos**	São Gonçalo do Rio Preto	S 18 09 23 W 43 22 09	23
18	**Itapanhoacanga**	Alvorada de Minas	S 18 47 11 W 43 26 49	42

N = number of individuals sampled per locality. N_ID: sampling point identification. PE = Parque Estadual (State Park), APA = Área de Proteção Ambiental.

Tadpoles were euthanized with benzocaine 20% and preserved in formalin 10% immediately after collection. They were stored in individual vials with locality and date information. Research and collection permits were obtained from Sisbio/ICMBio and the Instituto Estadual de Florestas (12813–1 and 085/06, respectively).

### Image analyses using GIS

In order to quantify human occupancy in the landscape we used RapidEye satellite images from 2010 made available by the Instituto Estadual de Florestas de Minas Gerais (IEF-MG). The RapidEye system is a constellation of five satellites with a unique ability to acquire high-resolution, large-area image data. The images have a spatial resolution of 6.5 m [49] and, after orthorectification, the resolution becomes 5 m per pixel of image, corresponding to a scale of 1:25000 [50].

We plotted UTM coordinates of sampling points (datum SAD69) over the RapidEye images and extracted two buffers with radius of 2.5 km and 368 m around them. The former corresponds to the distance between populations of *B*. *saxicola* beyond which they start to show significant genetic differentiation [42]. Thus, we considered this buffer as representative of the maximum area covered by local populations of the species. The smaller buffer represented the maximum core terrestrial habitat of frogs, according to a review of buffer zones around aquatic habitats [51]. In two instances the 2.5 km buffers of sampling points overlapped, so we merged them and pooled the tadpole samples of the sampling points.

**Fig 2:**
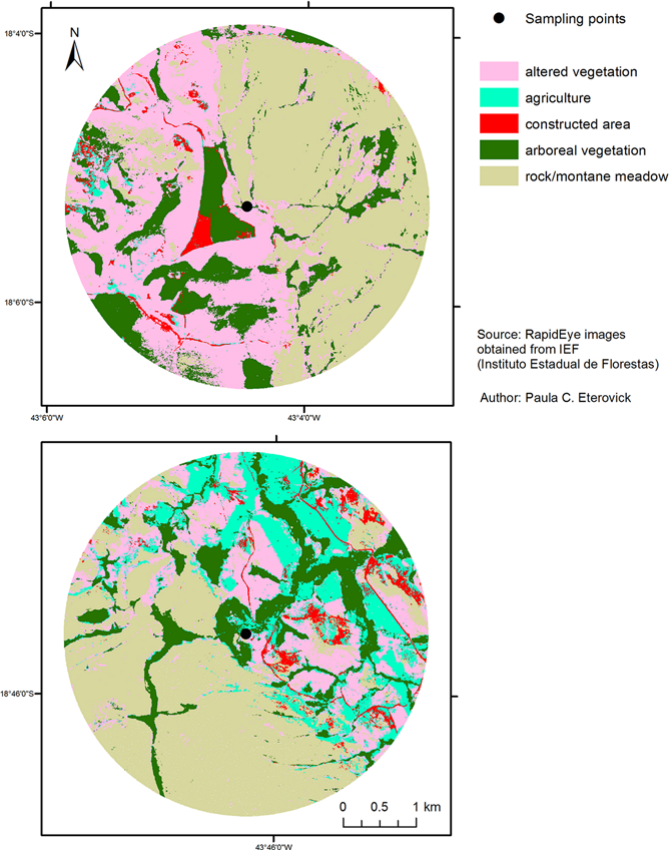
Examples of 2.5 km buffers used to quantify human occupancy (i.e., soil use classes) around two *B*. *saxicola* tadpole sampling points. Examples of 2.5 km buffers used to quantify human occupancy (i.e., soil use classes) around two *B*. *saxicola* tadpole sampling points: 17 (above) and 2 (below) (numbers are like in Table 1). The large constructed area close to sampling point 17 is a mine.

Within each buffer we estimated percentages of the total area comprised of each of a set of classes of soil cover, and then summed anthropogenic classes of soil cover (those with human influence) to obtain relative human occupancy in the landscape. We considered arboreal vegetation and rock/montane meadows to be natural habitats, and altered vegetation (pastures, areas occupied by introduced herbaceous plants), agriculture, and constructed areas (human settlements, roads, exposed soil around human settlements, mines) to be anthropogenic habitats (Fig. 2). We classified soil cover using the Interactive Supervised Classification function in the software ARCGIS. We classified separately and then grouped different shades of the same class (e.g., different crops in agriculture) and manually corrected instances in which the software was not able to split different landscape components (e.g., exposed soil around human settlements vs. naturally exposed rock). We then checked habitat classification in the field. Some of us (FSFL and PCE) have been conducting research throughout the Espinhaço Range since 1998, with more intensive and widespread sampling during 2006–2011, and know the area in detail. Although there was a 2-year interval between tadpole collection and map analyses, we are familiar with the localities included in this study and we know that no major changes in area used by humans occurred during this period that could influence our results.

We conducted all image analyses using the software ARCGIS at the Post Graduation Program Geografia—Análise da Informação Espacial at the Universidade Católica de Minas Gerais.

### Fluctuating asymmetry

**Fig 3:**
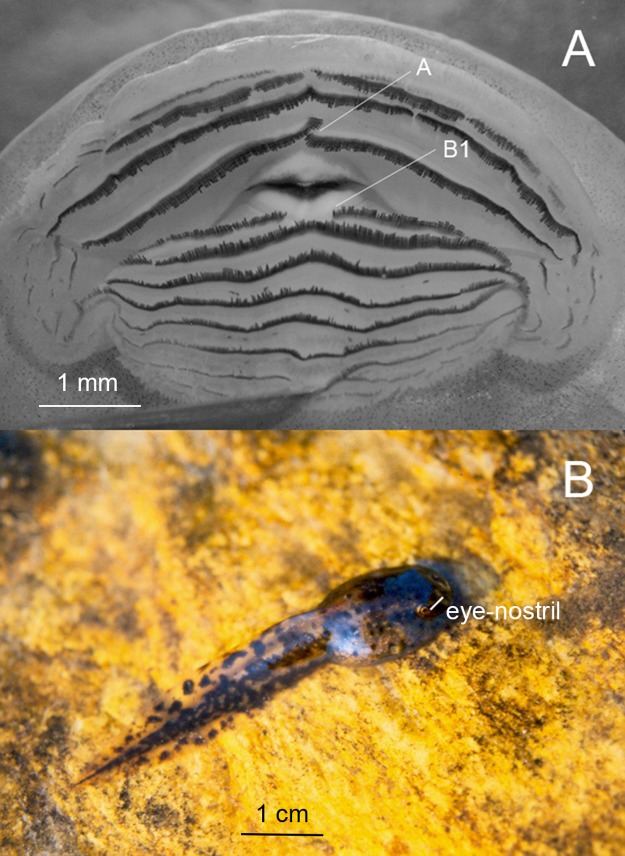
Traits used for quantification of fluctuating asymmetry in *Bokermannohyla saxicola* tadpoles. Traits used for quantification of fluctuating asymmetry in *Bokermannohyla saxicola* tadpoles included (A) denticle rows named A and B1 that correspond to the ones immediately anterior and caudal to the beak, respectively and (B) the closest distance from eye to nostril. Arrows indicate the central interval of denticle rows A and B1 in the tadpole’s mouth (A). Eye-nostril distance is indicated on a tadpole in its natural habitat (B).

To measure FA in the tadpoles, we selected the only traits that we considered we could measure/count with enough accuracy. Tadpole denticle rows are functional traits used in food acquisition [52, 53] and represent meristic traits with a large amount of FA, making them appropriate for DI studies [26]. We counted the number of denticles in tadpole oral discs in the last anterior row (called A) and the first posterior row (called B1) in relation to the beak (Fig. 3A). *Bokermannohyla saxicola* tadpoles have a labial tooth row formula (LTRF) 2(2)-3(3)/6–8(1), meaning that the rows we used to calculate FA are interrupted centrally, making it possible to unambiguously demarcate the right and left sides (Fig. 3A; for tadpole description and more illustrations, see [48]). We also used distance between eye and nostril (Fig. 3B), measured as the number of units in an ocular micrometer in a stereomicroscope (70 units corresponded to 1 mm at the 56X magnification we used). We performed all measurements twice using the “double blinded” method, in which each count on each side is made without knowledge of the results of other counts to avoid bias [17]. We randomized the order of tadpoles and measurements for each tadpole side to be measured. All data were collected by one person (LFFB), eliminating any possible observer effects.

### Statistical analyses

We performed FA analyses following the protocol proposed by [15]. We first searched for significant outliers according to Grubbs test statistics [54]. We eliminated individuals for which we detected significant outliers from subsequent analyses, because they may have been poorly preserved, making accurate counts difficult. Next, we performed the same test to eliminate outliers for differences between sides. Individuals that are too asymmetric may have malformations or physical damage, and thus be inappropriate subjects for FA measurement [15].

After the exclusion of outliers, we tested for effects of trait size on FA levels using Pearson correlations between the means of counts of both sides and the difference between them. Because there were correlations between trait size and FA, we used the asymmetry index FA_2_ of [26] in further analyses. This index is calculated by dividing the absolute value of the difference between measurements of right (R) and left (L) sides by the mean size of the sides (FA_2_ = |R-L|/(R+L)/2); this eliminates the effect of the individual’s size on its asymmetry level. In order to calculate the difference between sides we used the mean of the two replicate measurements to reduce the influence of measurement error.

We tested the original data (differences between right and left sides) for each variable for normality using tests for skewness and kurtosis [55]. If the absolute value of skewness (S) divided by its standard error (SES) is greater than 2 the coefficient is significant and indicates that the distribution is asymmetric [55], which could indicate directional asymmetry [15]. If the absolute value of kurtosis (K) divided by its standard error (SEK) is greater than 2, the coefficient is significant and indicates that the variable has longer tails than those for a normal distribution [49], which could indicate antisymmetry [15]. Our eye-nostril measurements were significantly skewed to the left, indicating that directional asymmetry was present, perhaps as a result of some subtle measurement bias. Therefore, only the tooth row counts (A and B1) were analysed further. We next used a mixed-model ANOVA with counts as dependent variables, and side, individual and their interaction as independent variables [15] to estimate mean squares (variances) for the side by individual interaction and for error (the variance within sides within individuals). We used these mean squares to calculate F statistics that compared the mean square for the side by individual interaction to the error mean square for each trait. When these are significant, and the underlying variation is normally distributed, they indicate that fluctuating asymmetry is significantly greater than measurement error, in other words that FA is a significant component of the overall variance of the character [15]. We also used the mean squares from these ANOVAs to calculate estimates of FA and measurement error for each character, following [15].

To test for significant diferences in levels of FA among sites, we calculated the differences between sides for each of the two sets of measurements (replicates) taken on each individual, then taking the absolute values of these differences and dividing by mean size of the character in that set of measurements and multiplying by 100 so that asymmetry was expressed as a percentage of character size. We then used the function glmer in R [56] to create generalized linear mixed models that related each response to the random effects of individuals only, and to the random effects of both individuals and localities. The models were created using identity link functions. We used the “anova” function in R to test the fit of the models including the effects of both localities and individuals against the fit of the models including only the effects of individuals; a significant increase in fit when locality was added indicated that FA differed among the localities we sampled. Finally, we conducted linear regression analyses to test for significant relationships between levels of size-corrected unsigned asymmetry (FA_2_) in tadpoles and the level of anthropogenic modification of the sampled landscapes. Except for the analyses specified as being carried out in R, we performed all statistical analyses in the software Systat [55].

## Results

We eliminated data on tooth row A from 13 individuals, on tooth row B1 from 8 individuals and on eye-nostril distance from 8 individuals from FA analyses as outliers, probably caused by measurement errors. In addition, we also eliminated data on tooth row A from 9 individuals, on tooth row B1 from 4 individuals and on eye-nostril distance from 11 individuals as asymmetry outliers, as defined by [15]. Thus, we proceeded with data from 227 individuals on tooth row A, 237 individuals on tooth row B1 and 230 individuals on eye-nostril distance.

Trait size was weakly but significantly correlated to FA (A: r = 0.3138, p < 0.0001; B1: r = 0.3012, p < 0.0001; eye-nostril distance: r = 0.2266, p < 0.0001). The distribution of differences between sides was not normal for eye-nostril distance (S/SES = -7.69, p < 0.05; K/SEK = 7.71, p < 0.05), indicating that directional asymmetry was present, so we did not use this trait in further analyses. Differences between sides did not differ significantly from the normal distribution for the other variables (A: S/SES = 1.91, p > 0.05; K/SEK = -0.45, p > 0.05; B1: S/SES = 0.80, p > 0.05; K/SEK = 0.15, p > 0.05).

Measurement errors for the normally-distributed tooth-row traits (A and B1) were far smaller than variation between sides (all p < 0.0005; Table 2). Measurement errors were on the order of 1% as large as the differences between sides attributable to FA (Table 2) and can be considered minor [15].

**Table 2:** Results of the two-way ANOVAs examining the relative magnitudes of FA plus antisymmetry and measurement error for the two traits of *Bokermannohyla saxicola* tadpoles (counts of denticles on either side of the mid-row gap in labial tooth rows A and B1) that showed normally distributed variation, and thus did not exhibit directional asymmetry.

Statistics	A	B1
MS_SIDES X INDIVIDUALS_	175.81	143.94
DF_SIDES x INDIVIDUALS_	213	223
MS_ERROR_	4.479	5.561
DF_ERROR_	428	448
F_SIDES X INDIVIDUALS_	39.253	25.883
P_SIDES X INDIVIDUALS_	<0.0005	<0.0005
ME5_a_	0.0819	0.0525
FA10_a_	7.386	6.638

The tests for side by individual interactions indicate whether levels of fluctuating asymmetry plus antisymmetry are significantly greater than levels of measurement error. FA10_a_ and ME5_a_ are estimates of population level fluctuating asymmetry and measurement error derived from the ANOVA results following [15].

Our comparison of full and reduced generalized linear mixed models of the random effects of individual and locality on levels of size-corrected, unsigned asymmetry showed that the random effects of locality (A: chi-squared = 170.65, df = 1, p < 0.0001; B1: chi-squared = 172.82, df = 1, p < 0.0001) were highly significant. This indicates that the unsigned asymmetry of both the A and B1 tooth rows differed significantly among our sampled collection localities.

**Fig 4:**
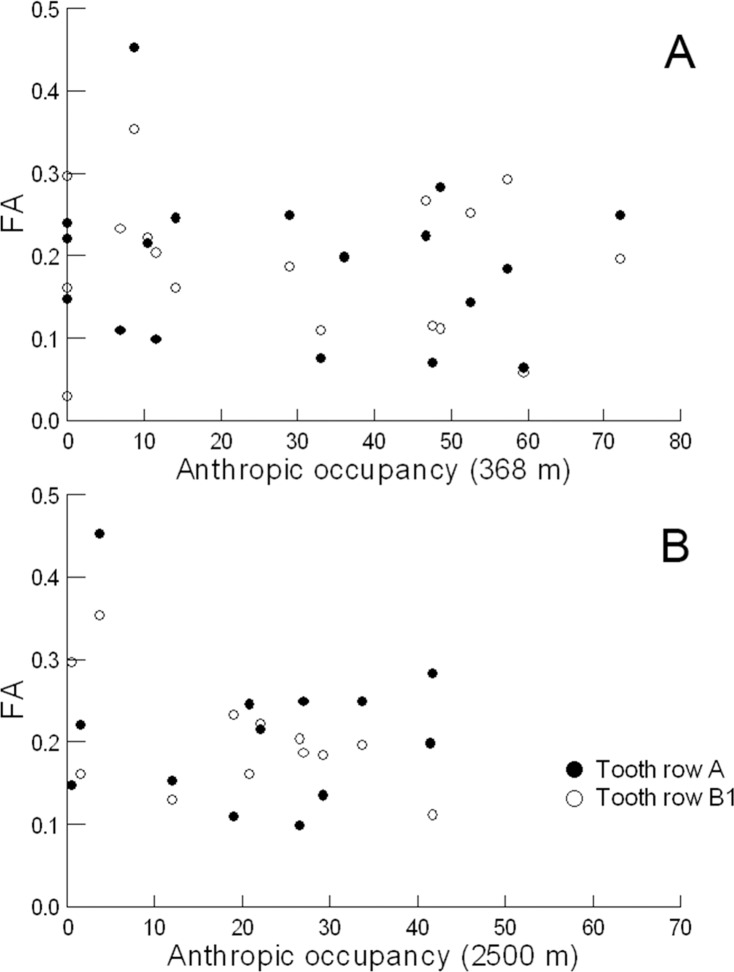
Relationships between FA levels in *B*. *saxicola* tadpoles and extent of anthropogenic modification of the landscape. Relationships between FA levels in *B*. *saxicola* tadpoles and extent of anthropogenic modification of the landscape (measured as percent cover of human soil use classes) considering buffers of (A) 368 m and (B) 2.5 km around tadpole sampling points.

We found no significant correlation between levels of unsigned asymmetry of either A or B1 and level of human impact. For the 368 m buffers, both relationships were not significant (A: R = -0.186, p = 0.459, df = 16; B1: R = -0.102, p = 0.687, df = 16; Fig. 4). When we examined potential correlations with degree of impact in the 2.5 km buffer, the buffers of points 5, 6, 7, and 14 overlapped, so we pooled them as a single buffer; for the same reason we also pooled points 3, 4, and 17 in another, leaving a total of 13 buffers. As with the level of impact immediately adjacent to bodies of water, the relationships between impact in these larger buffers surrounding our aquatic sites and unsigned asymmetry of tadpoles were not significant (A: R = -0.101, p = 0.744, df = 11; B1: R = -0.498, p = 0.083, df = 11; Fig. 4). To ensure that points with large variances caused by small sample sizes were not biasing our results towards a lack of statistical significance, we repeated the analyses excluding points with less than 5 individuals: points 8 and 13 for both buffer sizes and 3, 4, and 14 for the 368 m buffer only. All results remained not significant.

## Discussion

Our results did not confirm the hypothesis that *B*. *saxicola* from areas with a higher level of human occupancy would have elevated levels of FA in their larval stages caused by elevated levels of stress. This could reflect a lack of relationship between FA levels and one specific variable expected to index environmental stress; this has been found in some studies [57–59].

On the other hand, organisms can persist in adequate microhabitats even when these occur in unfavorable landscapes [60]. Therefore, despite the lack of correlation with habitat modification, the differences in levels of FA in *Bokermannohyla saxicola* tadpoles among sites might still reflect levels of environmental stress. Tadpoles are directly influenced by water quality where they develop [61]. Although human presence in the landscape ultimately influences water quality (e.g., through erosion, use of chemicals in agriculture), the relationship may not be consistent, because some human activities (and the ways they are performed) are likely to be more detrimental to water quality than others. Thus, measuring the quality of the aquatic habitat at a finer scale may be necessary to detect stressful developmental conditions experienced by tadpoles. Examining different human soil uses and the ways in which each activity is performed (e.g., types and amounts of chemicals used in agriculture) may also detect other stress conditions. However, because these variables varied widely among our sampling sites, our data were insufficient to test for the effects of each one separately on tadpole asymmetry. Studying the different degrees of impacts of human activities in natural habitats and how they affect amphibian populations is challenging, but is key to understand amphibian global declines [62].

Although there is no consensus on what specific water quality parameters would be optimal for amphibians, low oxygen concentrations, high temperatures, high levels of ammonia and low pH can all have negative effects on amphibian larval development [61]. Low pH has been shown to increase levels of FA in *Rana arvalis* populations [13]. High conductivity indicates high decomposition and is usually unfavorable to amphibians as well [63]. Other factors such as predator presence [64], type and quality of available food [65, 66], and density of competing tadpoles [67], are all important determinants of success at metamorphosis, and are potential causes of stress for tadpoles. Such biological factors may influence adult anuran and tadpole distribution [68, 69], and may be altered by human activities, although not necessarily in proportion to anthropogenic modification of the landscape. In addition, developmentally susceptible windows may exist and asymmetry outcomes are thus likely to vary depending on when individuals were exposed to stressful conditions [70].

The choice of traits to study FA is important and depends on how strongly their development is affected by noise and how noise and homeostatic processes are affected by environmental stimuli [71]. Many studies show significant differences in levels of FA among traits; those in which asymmetry may interfere with vital activities (such as locomotion) may tend to exhibit relatively lower asymmetry levels, for instance [71, 72]. Tadpole denticles could be considered in this category, because they are used in food acquisition [53, 73]. However, minor asymmetries in denticles may not interfere with food acquisition. Thus, the traits we used in this study may not be the best indicators of FA in tadpoles—although they were the only ones that we could measure with enough precision.

In attempts to associate FA with levels of environmental stress, genetic differences among populations are usually neglected, although they may alter responses to stress [21, 74, 75]. Geographical variations in asymmetry may occur because of genetic differentiation in morphology or developmental characteristics. Levels of FA might also be affected by levels of heterozygosity. Some studies have shown positive relationships between heterozygosity and developmental stability [21, 29]. This would lead to negative correlations between heterozygosity and FA among populations [76]. However, many studies have failed to find significant correlations between levels of asymmetry and heterozygosity [24, 60]. [74] propose that the evaluation of heterozygosity and genetic differentiation among local populations would add information to studies of geographical variation in FA levels. However, information on both aspects of genetics simultaneously is still not available for *B*. *saxicola* or any other anuran in the Espinhaço range. The effects of genetic variability on asymmetry may only be noticed under stressful environmental conditions [77].

Measurement of the areas used by humans in a landscape can be accomplished in a time and cost effective way by using satellite images. If such data were directly related to levels of impact on anurans it would provide a very useful tool to fight amphibian declines, allowing conservationists to act in the short term. FA is expected to reflect negative effects of a variety of human impacts on invertebrate and vertebrate species [14, 78]. However, from our study we cannot determine whether this technique is effective for tadpoles, at least at the scale and in the habitats we examined. Other indices of environmental stress, such as water quality, may be more effective for tadpoles. There are still questions to be answered, and future studies on the effects of environmental stress on amphibian FA should take into account different (and more specific) environmental variables, as well as other life stages and genetic variability within and among populations, about which there is still little information for Brazilian species [45].
